# Effect of Welding Heat Input on the Microstructure and Mechanical Properties of MIG-Welded Dissimilar Magnesium Alloy Joints

**DOI:** 10.3390/ma19102068

**Published:** 2026-05-15

**Authors:** Lingkai Jin, Xuhui Feng, Xiaoshan Tong, Wenjing Li, Jiaxin Huang, Jian Peng

**Affiliations:** 1 CRRC Zhuzhou Electric Locomotive Co., Ltd., Zhuzhou 412001, China; jlingkai@126.com (L.J.); 010200034543@crrcgc.cc (X.T.); 2 National Engineering Research Center for Magnesium Alloys, School of Materials Science and Engineering, Chongqing University, Chongqing 400044, China; fxh2001@163.com (X.F.); liwenjing02426@163.com (W.L.); 202209021019@cqu.edu.cn (J.H.)

**Keywords:** dissimilar magnesium alloy sheets, MIG welding, welding heat input, microstructure, mechanical properties

## Abstract

Welding is one of the key joining routes for expanding the engineering applications of dissimilar magnesium alloys. However, after experiencing rapid non-equilibrium heating and cooling cycles, the heat-affected zone (HAZ) of a welded joint tends to undergo grain coarsening as well as dissolution or agglomeration of precipitates, and therefore becomes the region most susceptible to failure. In this study, 3 mm thick sheets machined from AZ61A and AZ80A magnesium alloy hollow sections were joined by metal inert gas welding (MIG). Different ranges of welding heat input were obtained by combining multiple sets of welding parameters, in order to further tailor the HAZ of dissimilar magnesium alloy joints and achieve sound weld quality. The results showed that the joint exhibited the best overall mechanical performance at 523 J·mm^−1^, with an ultimate tensile strength, yield strength, and elongation of 292 MPa, 172 MPa, and 5.4%, respectively. All fractures occurred in the HAZ on the AZ61A side. Under this condition, the second phases in the HAZ were more finely and uniformly dispersed, with a volume fraction of 3.19%, an average size of 2.51 μm, and a minimum average grain size of 23.65 μm.

## 1. Introduction

To achieve energy conservation and emission reduction, magnesium alloys, as the lightest structural metallic materials, have been widely used in ground transportation, aerospace, and related fields [1,2,3,4,5]. High-quality joining of magnesium alloy components has become a critical prerequisite for their broader application, and joining between different magnesium alloy grades with dissimilar properties is particularly challenging. During welding, magnesium alloys are prone to coarse grains, cracks, and porosity, and their weldability is significantly poorer than that of aluminum alloys and steels [6,7,8]. Welding of thin-walled and thin-sheet magnesium alloy structures is especially difficult [9]. Therefore, reliable joining of medium- and high-strength AZ-series magnesium alloys remains an urgent issue to be addressed.

In addition to riveting, the main methods for joining dissimilar magnesium alloys include friction stir welding, laser welding, and arc welding [10]. Friction stir welding can readily produce welds with fine grains and without porosity [11,12,13], but its welding efficiency is relatively low. Laser welding offers high energy density, low heat input, a narrow heat-affected zone, and small welding distortion [14,15]; however, undercut is likely to occur, and the rapid cooling rate together with the small molten pool volume often leads to a relatively high porosity level [16]. MIG welding features high productivity and easy automation, and has thus become an important process for the efficient joining of magnesium alloys. MIG welding achieves a good balance between welding quality, production efficiency and process flexibility. Moreover, under optimized conditions, the joint efficiency of magnesium alloy joints produced by MIG welding can reach 80% to over 90%. Owing to the low melting point and pronounced evaporation tendency of magnesium alloys, MIG-welded joints are highly sensitive to heat input, which may induce grain coarsening as well as dissolution or agglomeration of second phases, thereby degrading mechanical performance. As a result, extensive studies have been carried out on MIG welding of magnesium alloys. Sun et al. [17] used AZ61A filler wire to MIG weld 5.5 mm thick AZ31B plates and found that increasing the welding current promoted grain coarsening in both the weld metal and the HAZ, whereas increasing the welding speed refined the grains and improved the joint properties. Wang [18] compared direct-current and alternating-current pulsed MIG welding and pointed out that the alternating-current pulsed mode can more precisely control heat input to the filler wire, thereby broadening the process window and improving parameter matching. Liu et al. [19] showed that increasing welding current reduced droplet size and accelerated droplet transfer frequency, progressing from globular transfer to projected transfer and then spray transfer; nevertheless, the intrinsically high heat input of MIG welding and the droplet transfer behavior remain key factors restricting the quality of magnesium alloy joints. Numerous studies have improved weld quality by optimizing the combination of current, voltage, and travel speed to regulate molten-pool homogeneity, stress state, and cooling conditions [20,21,22]. However, in the existing MIG welding literature on magnesium alloys, studies on MIG welding of medium-strength AZ61A and higher-strength AZ80A magnesium alloys are still scarce. The selection of this dissimilar combination was motivated by both engineering and scientific considerations. From an engineering perspective, AZ61A and AZ80A are the most widely used deformed magnesium alloys in the automotive and railway industries. In actual structural components, different regions often require different balances of strength, ductility, and cost. Therefore, establishing a reliable dissimilar joint between these two alloys has direct industrial relevance. From a scientific standpoint, although both alloys belong to the Mg–Al–Zn system, their aluminum contents differ significantly, resulting in distinct initial volume fractions of the β-Mg_17_Al_12_ precipitate. This difference renders the AZ61A–AZ80A system an ideal model for investigating how welding heat input influences the evolution of secondary phases in the heat-affected zone (HAZ) of dissimilar joints. Importantly, most existing studies on magnesium alloy MIG welding have focused on similar joints, while dissimilar combinations—particularly those involving alloys with markedly different aluminum contents and precipitate fractions—have received far less attention. The few reports on dissimilar welding are often limited to parameter optimization and rarely elucidate the mechanisms by which heat input affects secondary-phase evolution and grain structure in the HAZ of the weaker alloy. Previous studies typically treated the HAZ as a homogeneous region, overlooking the fact that in dissimilar joints, the HAZs on each side may have distinct initial microstructures.

To address this gap, the present study employed 3 mm thick extruded AZ61A and AZ80A magnesium alloy plates to systematically investigate the effects of varying heat input on the weld’s macroscopic morphology, microstructure, and mechanical properties. Furthermore, the study discusses the potential mechanisms by which welding heat input affects microstructural evolution and performance in the mechanically weakest region of the joint, providing an experimental basis for optimizing MIG welding processes of dissimilar magnesium alloys.

## 2. Materials and Experimental Procedures

Hollow thin-walled extruded profiles of AZ61A and AZ80A magnesium alloys were used as the base materials. Sheets with dimensions of 100 mm × 80 mm × 3 mm were obtained by laser cutting. AZ80A filler wire with a diameter of 1.2 mm was employed. The chemical compositions and mechanical properties of the AZ61A and AZ80A base materials are listed in Table 1.

**Figure 1:**
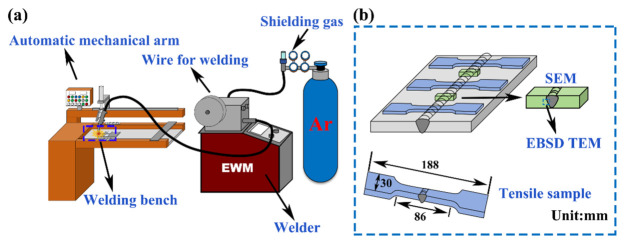
Experimental setup and sampling scheme for characterization: (**a**) Experimental setup; (**b**) Characterization sampling.

Welding was carried out on an EWM-MIG system (Ida New Technology Power Supply (Kunshan) Co., Ltd., Kunshan, China) mounted on a dedicated longitudinal seam welding platform, as shown in Figure 1a. The stand-off distance between the torch and the workpiece surface was 20 mm, and the torch inclination angle was 45°. High-purity argon (99.99%) was used as the shielding gas at a flow rate of 20 L·min^−1^. The welding voltage was fixed at 15.7 V. The welding currents were set at 140, 150, and 165 A, while the welding speeds were set at 3.1, 3.6, and 4.5 mm·s^−1^. The nine parameter combinations were grouped according to the welding-speed window, corresponding overall to low, medium, and high heat-input intervals, as listed in Table 2: low heat input (1#–3#), medium heat input (4#–6#), and high heat input (7#–9#). The welding heat input was calculated using:

(1) $$Q=\eta \frac{UI}{V}$$

where *Q* is the welding heat input, *U* is the welding voltage, *I* is the welding current, *V* is the welding speed, and *η* is the thermal efficiency coefficient, taken as 0.8.

The metallographic specimens were etched using a solution containing 4.2 g picric acid, 10 mL acetic acid, 70 mL absolute ethanol, and 10 mL deionized water. A field-emission scanning electron microscope (SEM, ZEISS Sigma 7800, Carl Zeiss (Shanghai) Management Co., Ltd., Shanghai, China) was used to characterize the second phases and other microstructural features. For EBSD characterization, the specimens were electropolished using AC_2_ polishing solution at −30 °C, 25 V, and 0.05–0.08 A for 1 min. Mechanical properties were measured on a universal testing machine at a crosshead speed of 2 mm·min^−1^. Three tensile specimens were prepared for each welding condition. The geometry of the tensile specimens and the sampling locations are shown in Figure 1b. Microhardness was measured across the base metal-HAZ-fusion zone path using a load of 0.1 kg, a dwell time of 15 s, and a step length of 0.5 mm.

**Figure 2:**
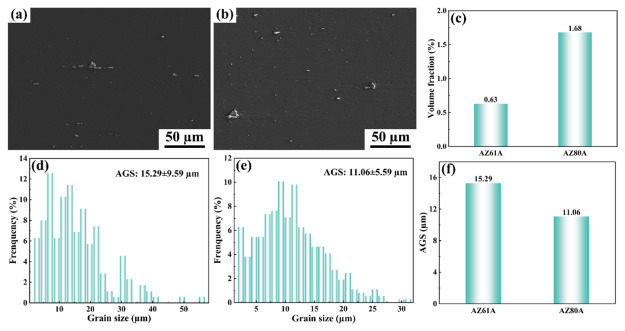
SEM images, second-phase volume fraction, and average grain size of the base materials: (**a**) AZ61A; (**b**) AZ80A; (**c**) second-phase volume fraction; (**d**) grain size distribution of AZ61A; (**e**) grain size distribution of AZ80A; (**f**) average grain size.

The microstructural characteristics of the as-received base materials prior to welding are shown in Figure 2. The average grain size and volume fraction of the second phase in AZ61A were 15.29 μm and 0.63%, respectively, whereas those in AZ80A were 11.06 μm and 1.68%, respectively.

## 3. Results

### 3.1. Mechanical Properties

**Figure 3:**
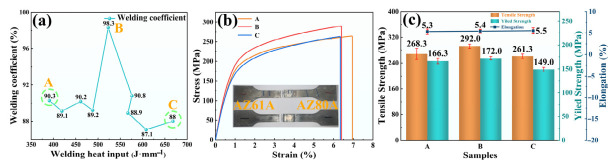
Room-temperature mechanical properties of the welded joints under different heat inputs: (**a**) joint efficiency; (**b**) stress–strain curves of representative joints; (**c**) variation trends of ultimate tensile strength, yield strength, and elongation of representative joints.

The room-temperature tensile results of the welded joints obtained under different heat inputs are shown in Figure 3. All tensile specimens fractured in the HAZ on the AZ61A side, indicating that this region was the weakest part of the joint. Within the heat-input range of 390–668 J·mm^−1^, the ultimate tensile strength of all joints remained higher than 85% of that of the AZ61A base metal. Among them, the joint produced at 523 J·mm^−1^ exhibited a joint efficiency of 98.3% and the best overall mechanical performance. To further clarify the effect of welding heat input on joint properties, three representative heat-input conditions, namely 391 J·mm^−1^ (A), 523 J·mm^−1^ (B), and 668 J·mm^−1^ (C), were selected for detailed comparison. The results show that the ultimate tensile strength, yield strength, and elongation first increased and then decreased with increasing welding heat input. At 523 J·mm^−1^, the joint reached an ultimate tensile strength of 292 MPa, a yield strength of 172 MPa, and an elongation of 5.4%. When the heat input was further increased to 668 J·mm^−1^, the ultimate tensile strength and yield strength decreased to 261.3 MPa and 149 MPa, respectively, while the elongation was 5.5%. These tensile results indicate that, within the heat-input range investigated here, a medium heat input is more favorable for obtaining joints with superior overall mechanical performance.

**Figure 4:**
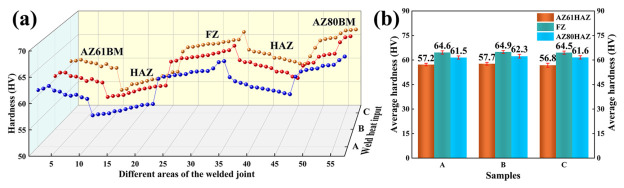
Microhardness of the welded joints under different heat inputs: (**a**) hardness distribution profile; (**b**) average microhardness values.

The microhardness distribution curves were plotted along the transverse direction, perpendicular to the weld, starting from the AZ61A base-metal side and extending to the AZ80A base-metal side, as shown in Figure 4. As can be seen from the curve profiles in Figure 4a, all joints exhibited a characteristic W-shaped hardness distribution under the three heat-input conditions, with the HAZ always showing the lowest hardness. The HAZ on the AZ61A side exhibited lower hardness than that on the AZ80A side, which is consistent with the tensile results showing fracture on the AZ61A side. Figure 4b quantitatively summarizes the average microhardness values in different regions under the three heat-input conditions. The average hardness values in the fusion zone (FZ) were 64.6, 64.9, and 64.5 HV, respectively, all higher than those of the HAZs on both sides. The average hardness values in the AZ61A-side HAZ were 57.2, 57.7, and 56.8 HV, respectively, which were the lowest within the joints. The corresponding values in the AZ80A-side HAZ were 61.5, 62.3, and 61.6 HV, lying between those of the FZ and the AZ61A-side HAZ.

### 3.2. Macroscopic Morphology

**Figure 5:**
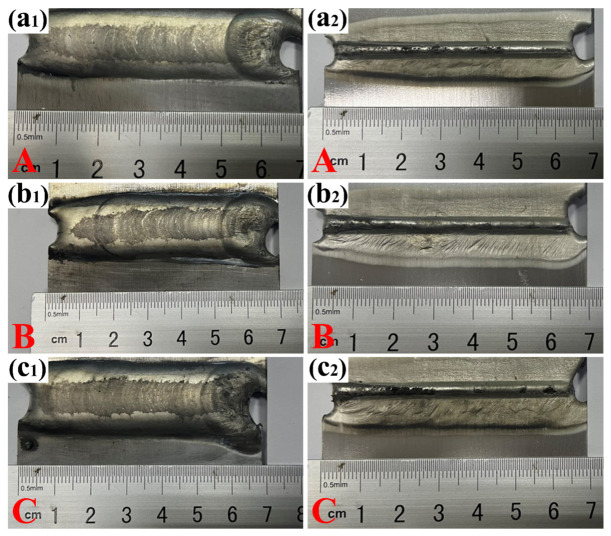
Macro-morphologies of joints welded under different heat inputs: 391 J·mm^−1^ (**a1**,**a2**); 523 J·mm^−1^ (**b1**,**b2**); 668 J·mm^−1^ (**c1**,**c2**).

The macro-morphologies of the joints welded under different heat inputs are shown in Figure 5. Under all three heat-input conditions, the weld beads were well formed, without obvious welding discontinuities such as incomplete welding or unfilled craters. Smooth transitions were obtained at both the arc-start and arc-stop regions, and no wire sticking was observed. With increasing welding heat input, the top and back bead appearances changed noticeably, and the fish-scale ripples on the weld surface became more uniform and denser.

Cross-sectional analysis was performed to systematically measure and examine the variations in reinforcement height, weld width, and penetration depth, as listed in Table 3. The results show that, as the heat input increased, the base metal was more fully melted, and the reinforcement height, weld width, and penetration depth all increased, with particularly significant increases in reinforcement height and weld width. When the heat input reached 668 J·mm^−1^, the reinforcement height and weld width reached maximum values of 3.15 mm and 22.2 mm, respectively. This can be attributed to the stronger heat-source action: increased heat input enhanced the arc energy and heating capacity, expanded the effective heat-source region, and consequently increased the weld width; the strengthened arc force promoted droplet detachment and increased the impact on the molten pool, favoring heat transfer in the thickness direction and thus increasing the penetration depth; thermal accumulation during continuous welding decreased the surface tension of the molten pool and strengthened the thermal effect of the arc on the base metal, thereby further promoting the increase in penetration depth and weld width; and the increase in heat input also raised the amount of melted filler wire per unit time, resulting in a larger deposited-metal volume per unit weld length and, hence, a marked increase in reinforcement height. However, excessively high heat input may also lead to adverse effects. For example, when the welding temperature exceeds the boiling point of the magnesium alloy, severe spatter may occur, which causes weld-metal loss and deteriorates the mechanical performance of the joint [23].

### 3.3. Microstructure

**Figure 6:**
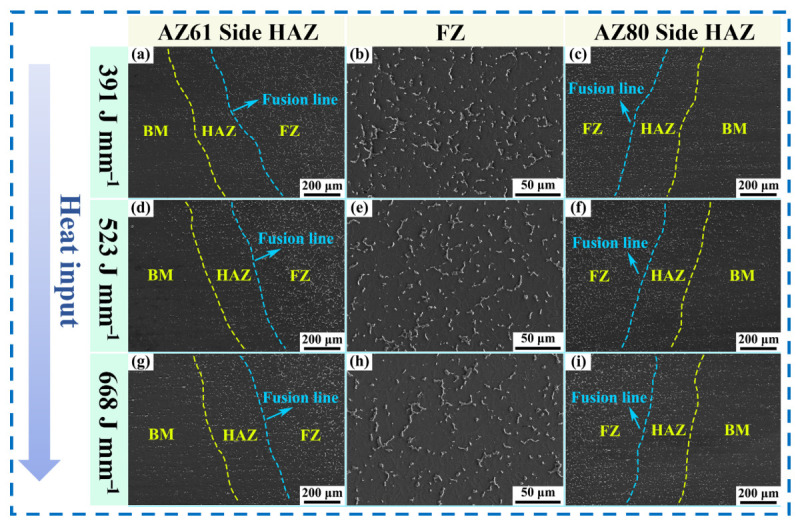
SEM images of the welded joints under different heat inputs: (**a**–**c**) 391 J·mm^−1^; (**d**–**f**) 523 J·mm^−1^; (**g**–**i**) 668 J·mm^−1^.

The morphologies of the second phases in the joints under different heat inputs are shown in Figure 6. In the FZ, the second phases were mainly distributed in network-like or chain-like morphologies and were significantly more abundant than those in the HAZ and the base metal (BM), being primarily distributed continuously along grain boundaries. In the HAZ, the amount of the second phase was relatively small and the particles were finer, indicating that this region underwent pronounced microstructural evolution under the welding thermal cycle. The quantity and morphology of the second phases in the BM were essentially the same as those in the as-received condition, indicating that the BM was only weakly affected by the welding thermal cycle.

**Figure 7:**
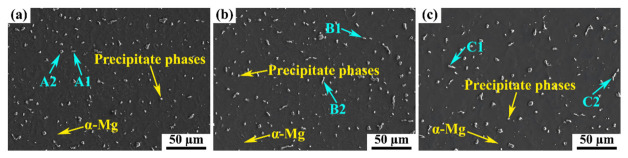
SEM images of the HAZ on the AZ61A side under different heat inputs: (**a**) 391 J·mm^−1^; (**b**) 523 J·mm^−1^; (**c**) 668 J·mm^−1^.

Since all joints fractured in the HAZ on the AZ61A side, this region was further characterized by SEM-EDS, as shown in Figure 7 and Table 4. The AZ61A-side HAZ consisted of a continuous α-Mg matrix containing a certain amount of bright precipitates dispersed within it, mainly in particulate and short rod-like morphologies. This indicates that, under the welding thermal cycle, the original second phases in the BM partially dissolved in the HAZ and then re-precipitated during cooling. Based on EDS analysis, the Mg-Al-rich phase can be inferred to be β-Mg17Al12, whereas the Al-Mn-rich phase can be inferred to be Al8Mn5. A comparison of the SEM morphologies under different heat inputs further shows that the welding heat input significantly affected the size and distribution state of the second phases in the AZ61A-side HAZ, thereby influencing the overall microstructural stability and mechanical properties of the joint.

**Figure 8:**
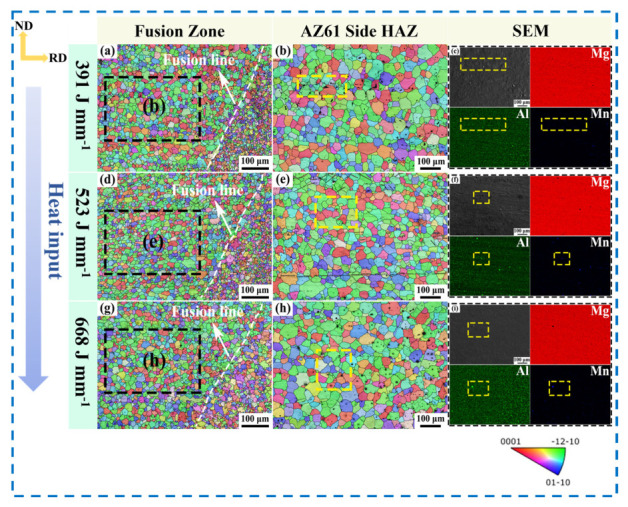
IPF maps of the joints under different welding heat inputs: (**a**) 391 J·mm^−1^ of the fusion zone; (**b**) The HAZ on the AZ61 side at 391 J·mm^−1^; (**c**) The yellow area in (**b**); (**d**) 523 J·mm^−1^ of the fusion zone; (**e**) The HAZ on the AZ61 side at 523 J·mm^−1^; (**f**) The yellow area in (**d**); (**g**) 668 J·mm^−1^ of the fusion zone; (**h**) The HAZ on the AZ61 side at 668 J·mm^−1^; (**i**) The yellow area in (**g**).

The inverse pole figure (IPF) maps and SEM images of the AZ61A side of the joints under different welding heat inputs are shown in Figure 8. A pronounced grain-size gradient was observed near the fusion line, and the welding heat input had a clear influence on the average grain size in the HAZ. At 391 J·mm^−1^, the grain-size distribution near the fusion line and in the HAZ was inhomogeneous, and locally coarse grains were observed. At 523 J·mm^−1^, the microstructure near the fusion line was the most homogeneous, and the grains in the AZ61A-side HAZ were the finest and most concentrated in size distribution. When the heat input was increased to 668 J·mm^−1^, grain coarsening reappeared in the HAZ. The corresponding SEM elemental maps indicate that, under 523 J·mm^−1^, the Al-Mn-rich phases were distributed more finely and uniformly, and the surrounding grains were correspondingly finer; in contrast, at 668 J·mm^−1^, the second phases exhibited a certain degree of coarsening.

## 4. Discussion

**Figure 9:**
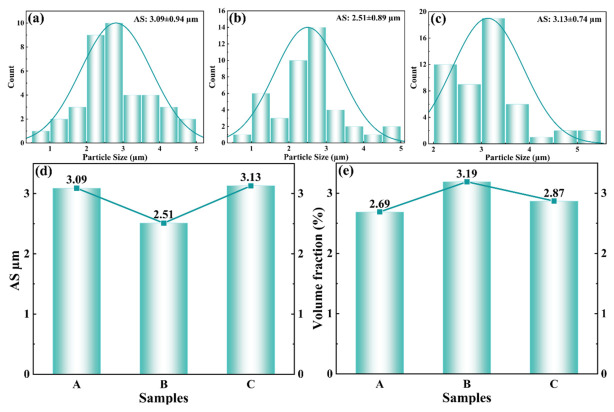
Average size and volume fraction of the second phase: (**a**) 391 J·mm^−1^; (**b**) 523 J·mm^−1^; (**c**) 668 J·mm^−1^; (**d**) statistics of average second-phase size; (**e**) statistics of second-phase volume fraction.

Second-phase particles can hinder the HAZ of AZ61 dislocation motion and thereby contribute to strengthening [24,25]; in addition, fine and dispersed particles can suppress the growth of recrystallized grains through grain-boundary pinning [26]. Variations in the amount, morphology, and spatial distribution of the second phase under different welding heat inputs directly lead to gradient changes in the microstructure and mechanical properties of the joint. Therefore, optimizing the welding heat input is beneficial for tailoring the size and distribution state of the second phases in the AZ61A-side HAZ, thereby improving the overall performance of the joint. Quantitative statistics for the second-phase particles in the AZ61A-side HAZ are shown in Figure 9. As the welding heat input increased from 391 to 523 J·mm^−1^, the volume fraction of the second phase increased from 2.69% to 3.19%, while its average size decreased from 3.09 μm to 2.51 μm. This indicates that, under a medium heat input, precipitation of the second phase in the HAZ was more sufficient, and the particles were finer and more uniformly dispersed. When the heat input was further increased to 668 J·mm^−1^, the volume fraction of the second phase decreased to 2.87%, whereas its average size increased to 3.13 μm, indicating that excessively high heat input is unfavorable for obtaining a fine and homogeneous second-phase distribution in the HAZ. This behavior may be related to changes in solute diffusion and precipitation conditions during the subsequent cooling process as a result of the higher peak temperature. The increase in molten-pool peak temperature also significantly raises the temperature in the adjacent HAZ, increases the solid solubility of Al in the α-Mg matrix, and thereby provides sufficient solute for the precipitation of Mg-Al and Al-Mn phases during cooling. Meanwhile, higher heat input is usually accompanied by a longer high-temperature dwell time and altered re-precipitation conditions of the second phase during subsequent cooling, which extend the diffusion time of alloying elements and allow Al atoms to migrate more fully to nucleation sites, thus promoting the formation of second phases. However, when the peak temperature becomes excessively high, the prolonged high-temperature residence time may cause particle coarsening or even local dissolution of the second phases.

**Figure 10:**
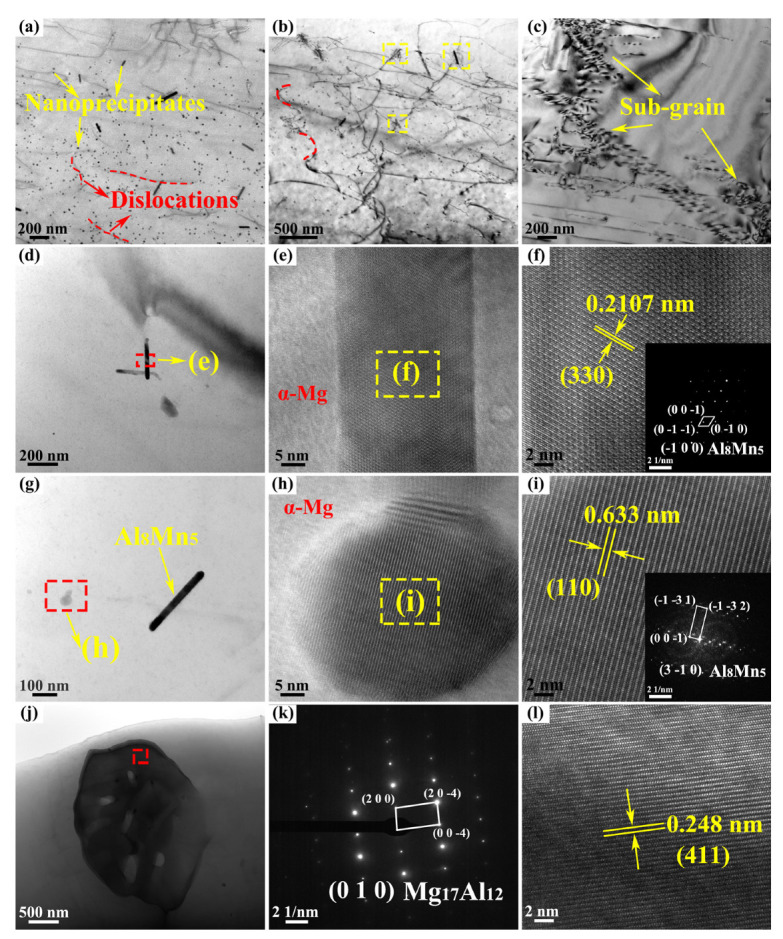
TEM images of the HAZ in the joint welded at 523 J·mm^−1^: (**a**–**c**) bright-field TEM images; (**e**) The red area in (**d**); (**d**,**g**,**j**) local high-magnification images of second phases; (**h**) The red area in (**g**); (**f**,**i**,**k**,**l**) corresponding SAED patterns, HRTEM images, and lattice-spacing measurements.

**Figure 11:**
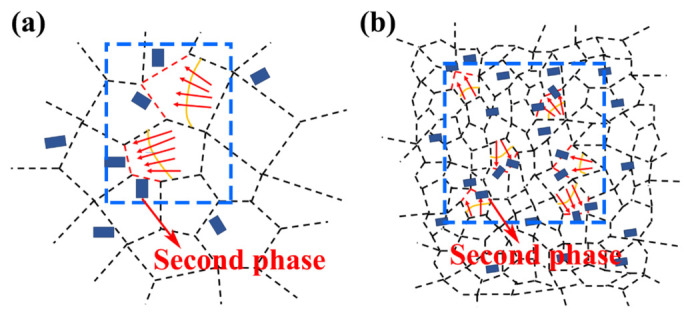
Effect of precipitate distribution and size on grain growth: (**a**) large precipitates; (**b**) fine and dispersed precipitates [27].

To further determine the crystal structure of the second phases in the AZ61A-side HAZ, transmission electron microscopy (TEM) was performed on the representative joint produced at 523 J·mm^−1^, and the results are shown in Figure 10. The selected-area electron diffraction (SAED) patterns and measured lattice spacings in the high-resolution images Figure 10f,i,k,l indicate that, besides the relatively large blocky β-Mg_17_Al_12_ phase, nanoscale Al_8_Mn_5_ particles with markedly different morphologies were also present in this region. The Al_8_Mn_5_ phase exhibited a bimodal morphology, namely rod-like and spherical particles. The rod-like particles were approximately 100–200 nm in length and 30–40 nm in width, whereas the spherical particles had an average diameter of about 10–20 nm and accounted for about 70% of the Al_8_Mn_5_ particles. As shown in Figure 8, regions containing more second-phase particles usually corresponded to finer and more homogeneous grains. The second-phase particles therefore played an important role in determining the grain size of the joint. On the one hand, uniformly dispersed particles are important for suppressing grain-boundary migration. The effect of the size and distribution of precipitates on grain growth is schematically illustrated in Figure 11: the smaller the particle size and the larger the volume fraction, the stronger the inhibition of grain growth and the finer the grains [27].

**Figure 12:**
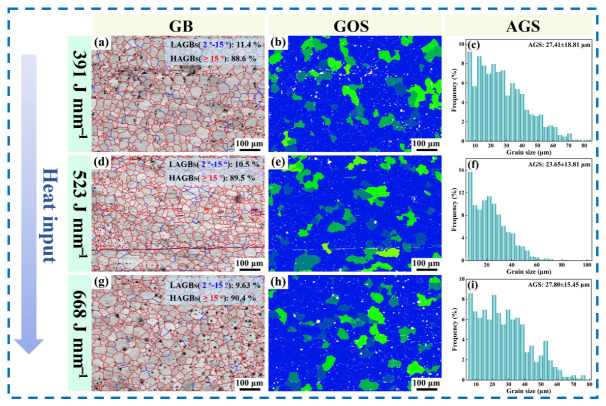
Grain-boundary characteristics, GOS, and grain size in the AZ61A-side HAZ under different welding heat inputs: (**a**–**c**) 391 J·mm^−1^; (**d**–**f**) 523 J·mm^−1^; (**g**–**i**) 668 J·mm^−1^.

On the other hand, relatively coarse second-phase particles may promote local recrystallization nucleation under certain conditions, whereas fine and uniformly dispersed particles enhance grain-boundary pinning, thereby effectively suppressing the growth of recrystallized grains and refining the microstructure. Grain refinement increases the grain-boundary area per unit volume, and the larger number of grain boundaries can impede dislocation motion and thus improve joint strength [28,29]. As shown in Figure 10b, obvious dislocation accumulation was observed around the interfaces of rod-like Al_8_Mn_5_ particles, while some dislocations bypassed the spherical Al_8_Mn_5_ precipitates, indicating that Al_8_Mn_5_ effectively hindered dislocation motion. At the same time, the fine and dispersed second-phase particles also suppressed grain-boundary migration. Therefore, the second phase improved the joint properties in two ways: by locally strengthening the material through impeding dislocation motion, and by alleviating grain coarsening through grain-boundary pinning. In addition, as shown in Figure 10c, a small number of subgrains were observed in the HAZ. Subgrain boundaries can also hinder dislocation motion and thus provide some subgrain strengthening. However, according to the EBSD statistics shown in Figure 12e, the subgrain area fractions under the three conditions were only 1.71%, 1.76%, and 1.44%, respectively, indicating that the contribution of subgrain strengthening to the joint strength was limited and clearly weaker than that of grain-refinement strengthening and second-phase strengthening.

Recrystallization is an important factor affecting grain size [30]. The low-angle and high-angle grain boundaries, grain orientation spread (GOS), and average grain size in the AZ61A-side HAZ under different welding heat inputs were statistically analyzed, as shown in Figure 12. GOS can be used to distinguish deformed grains from recrystallized grains [31]; recrystallized grains usually exhibit lower GOS values than deformed grains [32]. In the GOS maps, blue represents lower GOS values and thus a higher fraction of recrystallized grains. As can be seen from the figure, the HAZ underwent extensive recrystallization under the severe welding thermal cycles, and the area fraction of low-GOS regions increased with increasing heat input, indicating a rising proportion of recrystallized grains. The average grain size in the HAZ first decreased from 27.41 μm to a minimum of 23.65 μm and then increased again to 27.80 μm as the welding heat input continued to rise. Under all three welding heat inputs, the grain sizes were larger than those of the base metal. One reason is that, although sufficient recrystallization occurred in the HAZ as the temperature increased, the residual thermal effect subsequently caused static growth of the recrystallized grains. Another reason can be attributed to the influence of welding heat input on the second-phase particles. At the initial stage (heat input below the optimum value), only a limited amount of second-phase particles precipitated despite the welding thermal cycle. When the welding heat input exceeded the optimum value, although the cooling rate remained relatively high, the prolonged high-temperature residence time in the HAZ caused by the excessive heat input promoted coarsening of the second-phase particles, substantially increased grain-boundary mobility, diminished the pinning effect, and thus led to grain growth.

An increase in heat input leads to a higher peak temperature, a longer high-temperature dwell time, and a reduced cooling rate, which collectively influence solute diffusion and precipitation behavior in the HAZ. At relatively low heat input, insufficient thermal activation limits the dissolution and re-precipitation of second phases, resulting in a lower volume fraction of precipitates and limited strengthening. As the heat input increases to an intermediate level, enhanced diffusion promotes sufficient precipitation of fine and uniformly distributed second-phase particles, which effectively pin grain boundaries and suppress grain coarsening. This condition leads to the optimal combination of grain refinement and precipitation strengthening. However, when the heat input is excessively high, the prolonged exposure to elevated temperatures facilitates coarsening and partial dissolution of second phases, while also increasing grain-boundary mobility. As a result, grain coarsening becomes dominant, weakening the grain-boundary pinning effect and deteriorating mechanical properties. Therefore, the relationship between heat input and joint performance follows a typical “increase-then-decrease” trend, which reflects the competition between precipitation strengthening and grain coarsening. This mechanism is consistent with previous studies on magnesium alloy welding, but the present work further demonstrates that such a balance is particularly critical for dissimilar AZ61A–AZ80A joints, where the HAZ on the lower-strength side governs failure.

This conceptual framework provides a more general understanding of heat-input optimization in MIG welding of magnesium alloys and may serve as a basis for predicting microstructural evolution and mechanical performance under different welding conditions.

By correlating the ultimate tensile strength of the dissimilar magnesium alloy joints with grain coarsening in the HAZ under different welding heat inputs, a strong relationship was found between the two. Under low, medium, and high heat inputs, the ultimate tensile strengths were 268.3, 292, and 261.3 MPa, respectively, corresponding to reductions of 9.7%, 1.7%, and 12.01% relative to the base metal. The corresponding grain-coarsening degrees in the HAZ were 79.3%, 54.7%, and 81.8%, respectively. At 523 J·mm^−1^, the HAZ exhibited the smallest average grain size, 23.65 μm, and the lowest degree of coarsening; accordingly, the joint showed the best overall mechanical performance. According to the Hall–Petch relationship [33], a smaller average grain size generally leads to better mechanical properties of the welded joint. Overall, the improvement in joint properties is mainly associated with the combined effects of second-phase particles in the AZ61A-side HAZ, reduced grain coarsening, and alleviated local softening.

## 5. Conclusions

The effects of welding heat input on the microstructure and mechanical properties of MIG-welded dissimilar AZ61A-AZ80A magnesium alloy joints were systematically investigated. The main conclusions are as follows:

(1) Within the heat-input range of 390–668 J·mm^−1^, the ultimate tensile strength of all joints remained above 85% of that of the AZ61A base metal. Among them, the joint welded at 523 J·mm^−1^ exhibited the best overall mechanical performance, with an ultimate tensile strength of 292 MPa, a yield strength of 172 MPa, and an elongation of 5.4%. This can be attributed to an optimal balance between sufficient thermal energy to promote material plasticity and limited over-aging of strengthening phases, illustrating the critical role of heat input in controlling microstructural evolution and mechanical properties.
(2) All joints fractured in the HAZ on the AZ61A side, indicating that this region was the weakest part of the joint. Mg_17_Al_12_ precipitates and nanoscale Al_8_Mn_5_ particles were observed in the AZ61A-side HAZ. Under the medium heat-input condition, the second phase exhibited a higher volume fraction, smaller average size, and more dispersed distribution, which was beneficial for suppressing grain-boundary migration and improving joint properties. By contrast, excessively low heat input leads to insufficient precipitation and weak grain-boundary pinning, while excessively high heat input causes precipitate coarsening/dissolution and accelerated grain growth. Both extremes shift the balance away from the optimum, resulting in inferior mechanical properties.
(3) Welding heat input strongly influenced grain evolution in the AZ61A-side HAZ. At 523 J·mm^−1^, the HAZ exhibited the smallest average grain size (23.65 μm), corresponding to the optimal mechanical properties. This result reflects the interplay between dynamic recovery and limited grain coarsening: moderate heat input promotes continuous dynamic recrystallization and subgrain formation, leading to refined microstructure, whereas excessive heat input accelerates grain growth and deteriorates mechanical behavior.

## Figures and Tables

**Table 1:** Chemical compositions and mechanical properties of the base materials.

Base Metal	Chemical Composition/(%)							UTS /(MPa)	YS /(MPa)	EL /(%)
	Al	Zn	Mn	Fe	Si	Cu	Ni			
AZ61A	6.45	0.58	0.23	0.0023	0.0120	0.0019	0.00030	297.0	175	18.0
AZ80A	8.61	0.43	0.20	0.0037	0.0040	0.0018	0.00030	324.0	189	15.0

**Table 2:** Welding parameter combinations and heat input.

Sample	Welding Speed WS (mm/s)	Current I (A)	Voltage U (V)	Heat Input Q (J·mm^−1^)
1#	4.5	140	15.7	391
2#	4.5	150	15.7	419
3#	4.5	165	15.7	461
4#	3.6	140	15.7	488
5#	3.6	150	15.7	523
6#	3.6	165	15.7	576
7#	3.1	140	15.7	567
8#	3.1	150	15.7	608
9#	3.1	165	15.7	668

**Table 3:** Statistics of penetration depth, weld width, and reinforcement height.

Sample	Heat Input (J·mm^−1^)	Melting Width (mm)	Weld Bead Height (mm)	Penetration Depth (mm)
1#	391	18.6	1.73	4.92
5#	523	20.9	2.45	4.94
9#	668	22.2	3.15	5.00

**Table 4:** EDS results for the marked points in Figure 7.

Sample		Component (At.%)		
	Mg	Al	Mn	Zn
A1	21.7	51.5	26.7	0.1
A2	63.5	33.5	0.1	2.8
B1	35.0	45.9	18.7	0.4
B2	74.8	22.7	0.1	2.4
C1	20.3	51.6	28.1	0
C2	65.4	31.7	0.2	2.8

## Data Availability

The original contributions presented in this study are included in the article. Further inquiries can be directed to the corresponding author.
